# Antibiotic treatment with one single dose of gentamicin at admittance in addition to a β-lactam antibiotic in the treatment of community-acquired bloodstream infection with sepsis

**DOI:** 10.1371/journal.pone.0236864

**Published:** 2020-07-30

**Authors:** Karolina Liljedahl Prytz, Mårten Prag, Hans Fredlund, Anders Magnuson, Martin Sundqvist, Jan Källman

**Affiliations:** 1 Department of Infectious Diseases, Faculty of Medicine and Health, Örebro University, Örebro, Sweden; 2 Department of Clinical Microbiology, Faculty of Medicine and Health, Örebro University, Örebro, Sweden; 3 Clinical Epidemiology and Biostatistics, School of Medical Sciences, Örebro University, Örebro, Sweden; University of Western Australia, AUSTRALIA

## Abstract

**Background:**

Combination therapy in the treatment of sepsis, especially the value of combining a β-Lactam antibiotic with an aminoglycoside, has been discussed. This retrospective cohort study including patients with sepsis or septic shock aimed to investigate whether one single dose of gentamicin at admittance (SGA) added to β-Lactam antibiotic could result in a lower risk of mortality than β-Lactam monotherapy, without exposing the patient to the risk of nephrotoxicity.

**Methods and findings:**

All patients with positive blood cultures were evaluated for participation (n = 1318). After retrospective medical chart review, a group of patients with community-acquired sepsis with positive blood cultures who received β-Lactam antibiotic with or without the addition of SGA (n = 399) were included for the analysis. Mean age was 74.6 yrs. (range 19–98) with 216 (54%) males. Sequential Organ Failure Assessment score (SOFA score) median was 3 (interquartile range [IQR] 2–5) and the median Charlson Comorbidity Index for the whole group was 2 (IQR 1–3). Sixty-seven (67) patients (17%) had septic shock. The 28-day mortality in the combination therapy group was 10% (20 of 197) and in the monotherapy group 22% (45 of 202), adjusted HR 3.5 (95% CI (1.9–6.2), *p* = < 0.001. No significant difference in incidence of acute kidney injury (AKI) was detected.

**Conclusion:**

This retrospective observational study including patients with community-acquired sepsis or septic shock and positive blood cultures, who meet Sepsis-3 criteria, shows that the addition of one single dose of gentamicin to β-lactam treatment at admittance was associated with a decreased risk of mortality and was not associated with AKI. This antibiotic regime may be an alternative to broad-spectrum antibiotic treatment of community-acquired sepsis. Further prospective studies are warranted to confirm these results.

## Introduction

Sepsis is a life-threatening organ dysfunction caused by a dysregulated host response to infection [1]. The syndrome has a high mortality (10–56%) even if treated according to established guidelines [1–8]. Despite the lack of new antibiotics in the treatment of sepsis, a decline in 28-day mortality has been reported [9, 10]. Different explanations suggested for this have included differences in sepsis definitions, study populations, study settings, or times to treatment [11–15]. Appropriate choice of antibiotic treatment has been proven to significantly reduce mortality [16].

According to the Surviving Sepsis campaign [8], aminoglycosides can be administered as a once-daily dose repeated over several days together with a β-Lactam in patients with septic shock (defined by Sepsis-3 criteria) [1]. By adding gentamicin to a β-Lactam antibiotic, the antibiotic spectrum is expanded and the bacteria are attacked in two different ways, thus accelerating the clearance of pathogens [8, 13, 17, 18]. This improved bacterial kill rate is probably most important during the first hours after presentation [19].

In Sweden, one single dose of an aminoglycoside at admission (SGA) without subsequent doses after admission, in combination with a β-lactam antibiotic (over several days) has been a common clinical approach to patients presenting symptoms consistent with sepsis at the emergency department (ED) [15]. Re-evaluation of the antibiotic treatment is made within 24 hours. A similar antibiotic strategy is used in the Netherlands [20]. The rationale for this is good coverage against the most common bacteria causing pneumonia or pyelitis. Dosage is based on body weight, 5 to 7 mg/kg gentamicin and it is administered together with the first β-Lactam dose at the ED immediately after blood cultures have been retrieved from the patient. The SGA is administered independent of kidney function. Although the concept of SGA has been used in Sweden for many years, the strategy has not been substantially investigated regarding 28-day mortality and the risk of AKI. Recent studies, many included in a meta-analysis performed by Paul et al [11], has not shown decreased mortality but rather elevated number of side effects such as nephrotoxicity [11]. Benefits could however, be observed in patients with septic shock [12]. Importantly the meta-analysis did not include any articles with the SGA-concept described above but gentamicin/aminoglycoside administration over several days, either administered once daily (‘single-dose regime’) or as multiple doses per day [15]

The primary aim of this study was to evaluate the effect of the addition of one SGA given with the first dose of β-Lactam antibiotic compared with β-Lactam monotherapy on all-cause mortality at 28 days in the treatment of patients with sepsis.

The secondary aim was to study whether SGA was associated with AKI.

## Methods and material

### Study design, settings, and patients

Patients with positive blood cultures were retrieved from the laboratory information system (Mikro96) at the Department of Clinical Microbiology, Örebro University Hospital. From January 2011 to December 2012, 1318 individual positive blood cultures were found. The cultures had been drawn from adult patients at the emergency department of the Örebro University Hospital, Örebro, Sweden and two nearby affiliated hospitals. These defined the selected cohort for further evaluation (n = 1318). Following patients were excluded: candidemia (n = 7), contamination (n = 375), nosocomial infections (n = 326), non-β-Lactam antibiotic treatment (n = 150) and/or not fulfilling the Sepsis-3 criteria (n = 61). This study focused on patients admitted to the hospital directly from the emergency room treated with a β-Lactam antibiotic with or without the addition of one single dose of gentamicin at admittance based on bodyweight (5–7 mg/kg). Organisms commonly recovered from the environment or the skin (mainly coagulase-negative staphylococci) were assessed as contaminants except when clinical findings (i.e., results of cultures from other body sites or two or more positive sets) indicated a high probability of bloodstream infection. Community-acquired infections were defined as infections manifesting and diagnosed within 48 hours of admission to the hospital, without any nearby encounter with healthcare. Sepsis-3 was defined as organ dysfunction characterized by a rise of ≥2 in total SOFA score due to a dysregulated host response to infection, and septic shock as Sepsis 3 plus a lactate >2 mmol/L and vasopressor treatment needed to keep the mean arterial pressure ≥65 mmHg, despite adequate fluid resuscitation [1, 21].

**Fig 1:**
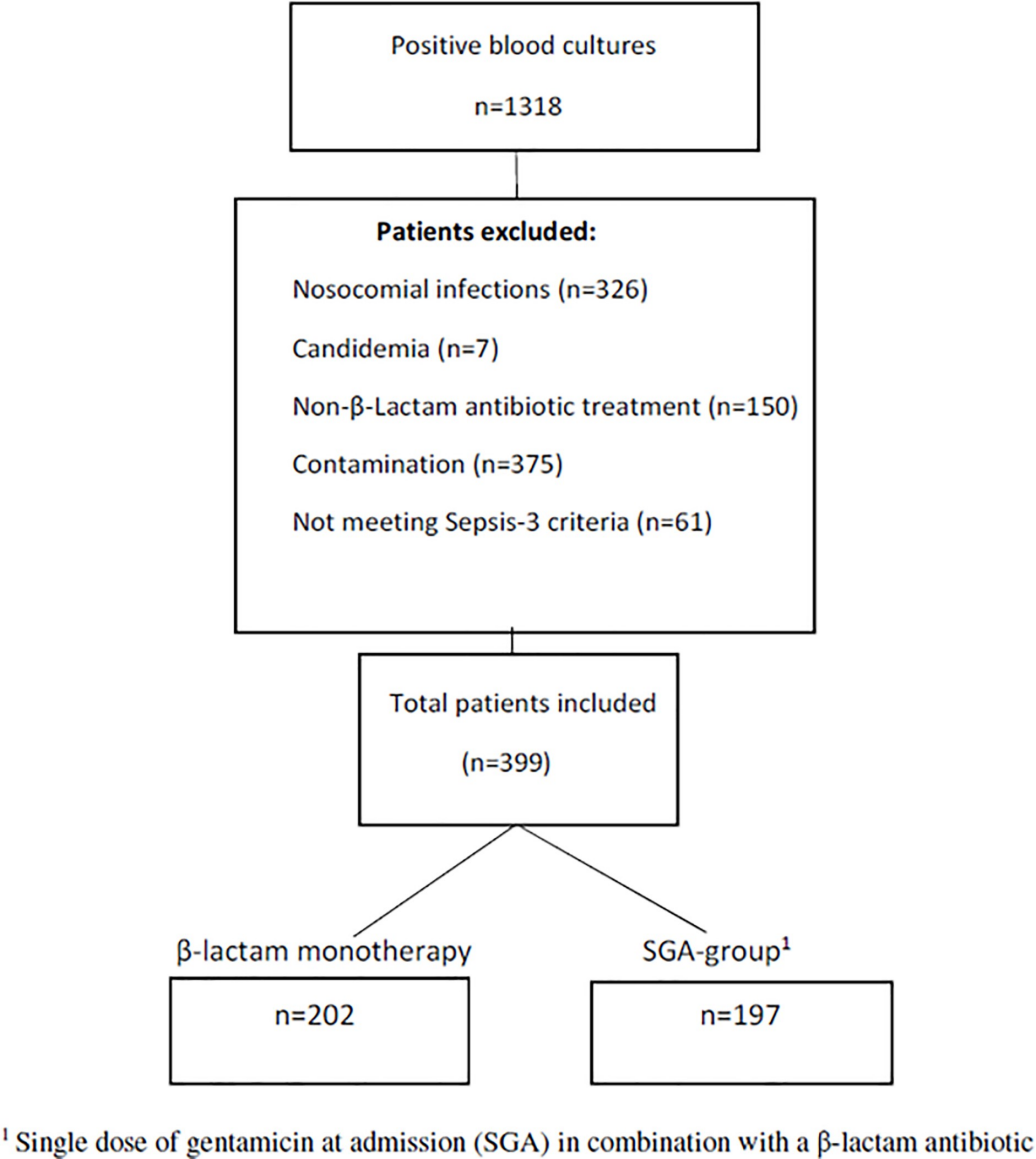
Inclusion flow chart.

After exclusion, 399 individual cases were studied (Fig 1) out of which two cohorts were defined:

1. 202 patients who received monotherapy with a β-Lactam antibiotic on admittance to the hospital, and
2. 197 patients who received the addition of SGA in combination with a β-Lactam antibiotic.

Patients with symptoms of sepsis or septic shock with negative blood cultures were not under consideration in the present study since the main purpose was to examine the outcome of different antibiotic strategies in relation to microbiological findings.

### Data sources/measurements

Patient characteristics, comorbidity, laboratory results at admission, time of death (if applicable), and choice of antibiotics were retrieved from each patient’s hospital record. Antibiotics were grouped into broad-spectrum β-lactams (i.e. with antipseudomonal effect) or other β-lactams [22]. Baseline characteristics included age, sex, NEWS2 score [23–25], isolated pathogens, and parameters for calculating the SOFA score [1, 26, 27]. Previously accepted formulas were used to estimate mean arterial pressure (MAP) and PaO_2_/FIO_2_ [21, 28, 29]. NEWS2 and SOFA scores were measured only once at admittance to the emergency department. No follow up measurement for trends was possible. Comorbidity was assessed using the Charlson Comorbidity Index [30]. Laboratory results included platelet blood count, s-bilirubin, s-lactate and s-blood platelets. Above this, information about immunosuppression, admittance to the Intensive Care Unit (ICU), inotropic drugs administration, and comorbidities were retrieved.

S-creatinine was registered, if available, at four times: T0 (baseline, i.e., before admission within 6 months), T1 (at admission), T2 (day 2–5), and T3 (day 7–14) [20, 31]. AKI at admission was defined by the Risk, Injury, Failure, Loss, End stage renal disease classification (RIFLE) [32]. The definitions of RIFLE are Risk—creatinine 1.5 times T0; Injury—creatinine 2.0 times T0; failure—creatinine 3.0 times T0; Loss—complete loss of renal function > 4 weeks; and End-stage kidney disease complete loss of kidney function >3 months. Patients with previously chronic kidney disease (CKD) were excluded in calculations of the risk for AKI due to difficulty to evaluate the change in s-creatinine in these patients. CKD was defined according to current international guidelines as decreased kidney function measured in glomerular filtration rate (GFR) of <60mL per 1.73m2, of at least 3 months duration [33]. Neutropenia (defined as neutrophil granulocyte count <0.5 × 10^9^/L), chemotherapy, steroid treatment (equal of Prednisolone ≥ 20 mg/day), and immune modulatory treatment were considered immune suppression.

One investigator (KLP) collected all variables, but matters were discussed within the research group. Inappropriate antimicrobial coverage was defined as use of an antimicrobial agent, to which a pathogen was resistant according to the SIR system, using European Committee on Antimicrobial Susceptibility Testing (EUCAST) clinical breakpoints as defined at the time of the study [34].

### Blood cultures

At the time of the study the recommendation at the ED was to aseptically collect venous blood (15–20 ml) in two sets of blood cultures, one aerobic and one anaerobic bottle each (total 30–40 ml). Blood samples were taken from at least one peripheral venous access and cultures were incubated using the BACTEC FX system (Becton Dickinson, Franklin Lakes, NJ, USA). Genus and species of microorganisms were determined by standard methods used at the laboratory at the time of the study. Susceptibility testing was performed using disk diffusion according to EUCAST methodology and interpreted according to EUCAST breakpoints valid at the time of the study.

### Missing data

In two of the departments s-bilirubin and platelet count were not routinely measured. This led to 72 patients with incomplete SOFA score.

### Statistical methods

Continuous variables were evaluated with unpaired *t*-test and categorical variables with Chi-square or Fisher exact test as appropriate. Kaplan-Meier and Cox regression were used to visualize and compare time to 28 days mortality between the SGA and β-lactam monotherapy groups. All patients had complete follow-up. The Cox regression was adjusted for potential confounding variables age, Charlson comorbidity index, SOFA score, and NEWS2 score modelled on continuous scale and sex, broad or narrow-spectrum of antibiotics and immunosuppression on categorical variables. As a sensitivity analyses the adjusted model was also estimated after categorizing the continues variables but as the finding of the main hypothesis were very similar the results were not presented. For further details, see supplement 2 in S2 File. Subgroup analyses were performed by stratifying on 1) sepsis and septic shock 2) known and unknown foci of infection 3) type of bacteria. Cox regression gives hazard ratios (HR) supplemented with 95% confidence intervals (CIs) as association measures. The adjusted model was also evaluated with an IPTW (Inverse Probability weighted) logistic regression model, using a probit model with the same independent variables as above to estimates the weights. A random intercept linear mixed model was used to evaluate the secondary outcome, serum creatinine. Study groups, times (T0, T1, T2, T3), and the interaction groups × time were fixed factors, and the model’s estimated marginal means were reported with 95% CIs. The mixed model is similar to analysis of variance (ANOVA) for repeated measurements, but it has the advantage that patients with missing data are included on the assumption that the missing data are random. The analyses were adjusted for the same potential confounding variables as above and stratified on AKI (yes/no) status at T1. As sensitivity analyses, serum creatinine on log_10_ scale was also evaluated but as the statistically significant findings were not altered, these results were not presented.

We performed no sample size calculation prior to the study. After including 50 patients, an estimation was made that gave an approximation of number of patients needed to be included to have a chance of detecting significant results. All reported *p* values were two-tailed and a *p* value of <0.05 was considered statistically significant. All statistical analyses were performed using STATA release 14 (STATA Corp. TX, USA) and SPSS version 22 (IBM Corp. Armonk, NY, USA).

### Ethics

Ethical Approval from Uppsala Ethical Review Board was retrieved December 2013 (EPN 2013/451, Ö63-13). Informed consent was not necessary according to the Ethical Review Board since it is a retrospective observational study.

## Results

### Patients and cohort demographics

A total of 1318 patients with positive blood culture was identified. After thorough evaluation of the patients’ medical charts and assessment of the exclusion criteria, 399 patients were included for further analysis (Fig 1). Mean age was 74.6 and 54% males. The median SOFA score was 3 and median Charlson Comorbidity Index was 2. The SGA-group had a statistically significant higher mean age and higher mean NEWS2 score (Table 1) than the β-Lactam monotherapy group. *Pseudomonas aeruginosa*, *Enterobacter* spp. and *Serratia marcescens* were found in 18 separate patients, where only 4 patients had some sort of immunosuppression.

**Table 1:** Baseline characteristics.

	Total (n = 399)	β-lactam monotherapy (n = 202)	SGA-group^1^ (n = 197)	*p*
Age—mean (SD)	74.6 (14.5)	72.9 (16.0)	76.4 (12.5)	0.014
Male–no. (%)	216 (54%)	102 (50%)	114 (58%)	0.14
**Comorbidity**				
Charlson score–median (IQR)	2.0 (1–3)	2.0 (0–3)	2.0 (1–3)	0.48
**Kidney function evaluation**				
Chronic kidney disease–no. (%)	107 (27%)	56 (28%)	51 (26%)	0.68
AKI at T_1_^2^ (n = 292)	92 (32%)	41 (28%)	51 (35%)	0.208
**Immune suppression**–no. (%)	51 (13%)	31 (15%)	20 (10%)	0.12
Neutropenia–no.	13	9	4	
Steroid treatment—no.	8	7	1	
Immunomodulating agents–no.	28	14	14	
Chemotherapy–no.	2	1	1	
**Sepsis severity**				
Septic shock–no. (%)	67 (17%)	29 (14%)	38 (19%)	0.19
SOFA-score—median (IQR)	3.0 (2–5)	3.0 (2–5)	3.0 (2–5)	0.34
NEWS2-score—median (IQR)	7.0 (5–9)	6.0 (4–8)	7.0 (6–10)	<0.001

^1^ SGA in combination with a β-lactam antibiotic.

^2^ Patients with CKD was excluded in this calculation.

Septic shock was diagnosed in 67 patients (17%). These patients were all treated in the ICU where inotropic drug support was initiated. From the retrospective chart review, 161 patients were assessed as having an obvious focus of infection and 238 an unknown focus.

Diagnoses were pneumonia, pyelonephritis, wound infection, erysipelas, endocarditis, abdominal infection, meningitis, cholecystitis and throat infection.

### Mortality

**Fig 2:**
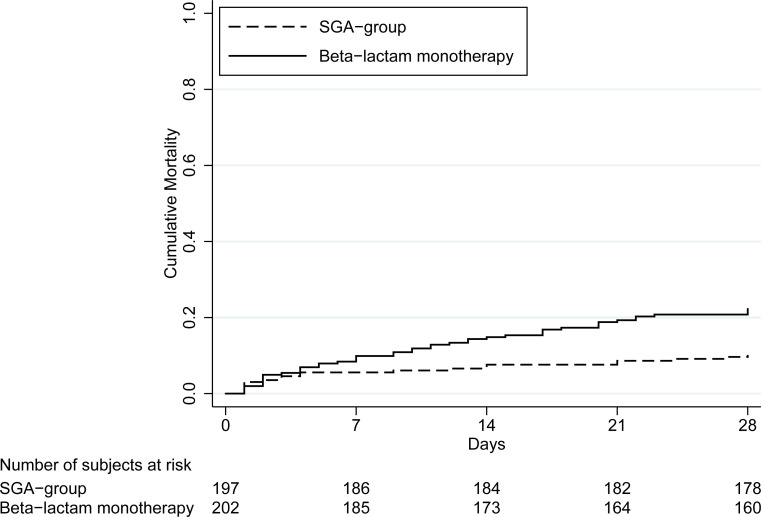
Kaplan Meier curve illustrating the significant difference in mortality between the monotherapy group (22%, 45/202) and SGA (10%, 20/197).

Overall, the 28-day mortality was 16% (65 of 399). The β-Lactam monotherapy group displayed a significant higher mortality (22%, 45/202) than the SGA-group (10%, 20/197), adjusted HR 3.5 (95% CI 1.9–6.2), *p <* 0.001. This is illustrated in a Kaplan-Meier curve (Fig 2). IPTW model showed adjusted OR 4.3 (95% CI 2.0–9.4), *p*<0.001. Among patients with sepsis adjusted HR was 3.4 (95% CI 1.8–6.5), *p* < 0.001 and in the septic shock subgroup HR 4.3 (95% CI 1.0–17.7), *p* = 0.043. In the subgroup of patients with obvious focus of infection the unadjusted HR was 3.4 (95% CI 1.1–10.4), *p* = 0.036 and adjusted HR 4.4 (95% CI 1.3–15.1), *p* = 0.019. Among patients with unknown focus of infection unadjusted HR was 2.0 (95% CI 1.1–3.6), *p* = 0.023 and adjusted HR 3.4 (95% CI 1.8–6.6), *p*<0.001.

### Different bacteria and mortality

Bacterial agents in blood cultures were grouped as shown in Table 2. Most common pathogens were *E*. *coli* (22%), followed by *S*. *aureus* (21%) and *S*. *pneumoniae* (16%). For further details, see supplement 1 in S1 File. Patients with infections caused by *E*. *coli* and *S*. *aureus* showed a significant difference in mortality between β -Lactam monotherapy and SGA-group in the adjusted models.

**Table 2:** Microbiology findings in blood cultures, susceptibility to administered antibiotic regime and 28-day mortality.

			β-lactam monotherapy				SGA-group^1^				β-Lactam monotherapy vs SGA groups			
	Total n = 399	Mortality (%)	Sensitive	Resistant	Total	Mortality (%)	Sensitive	Resistant	Total	Mortality (%)	Unadjusted Cox regression		Adjusted^2^ Cox regression	
											HR (95% CI)	*p*	HR (95% CI)	*p*
**Streptococci**														
Alpha-hemolytic streptococci	18	5 (28)	9	0	9	3 (33)	9	0	9	2 (22)				
Beta-hemolytic streptococci	26	6 (23)	12	0	12	3 (25)	14	0	14	3 (21)				
*S*. *pneumoniae*	55	6 (11)	23	0	23	2 (9)	31	1	32	4 (13)	0.7 (0.1–3.7)	0.66	1.8 (0.1–26.7)	0.68
**Enterococcus**														
*E*. *faecalis*	8	0	1	0	1	0	4	3	7	0				
**Staphylococcus**														
*S*. *aureus*	74	16 (22)	39	1	40	13 (33)	34	0	34	3 (9)	4.0 (1.1–14.0)	0.031	12.0 (2.6–56.0)	0.002
Coagulase negative staphylococci	9	0	5	1	6	0	2	1	3	0				
**Enterobacterales**														
*Escherichia coli*	126	16 (13)	65	5	70	11 (16)	56	0	56	5 (9)	1.8 (0.6–5.3)	0.26	4.1 (1.1–14.9)	0.034
*Klebsiella* spp.	29	4 (14)	13	1	14	3 (21)	15	0	15	1 (7)				
Other*	15	2 (13)	7	0	7	2 (29)	8	0	8	0 (0)				
*Pseudomonas aeruginosa*	12	5(41)	2	2	4	3(75)	8	0	8	2(25)				
Anaerobic bacteria**	12	3(25)	2	5	7	3(43)	4	1	5	0				
Other***	15	2(13)	7	2	9	2(22)	6	0	6	0				
**Total**	399	65 (16)	185	17	202	45 (16.2)	191	6	197	20 (10.2)				

^1^ SGA in combination with a β-lactam antibiotic.

^2^ Adjusted for age, Charlson comorbidity score, SOFA-score and NEWS2-score as continuous variables and sex, broad- or narrow antibiotic spectrum and immunosuppression as categorical variables.

*Enterobacter spp. n = 5, *Proteus mirabilis* n = 8, *Serratia Marcescens* n = 1.

**Bacteroides fragilis n = 8, Anaerobic Gram positive cocci n = 2, *Clostridium* spp. n = 1, *Fusobacterium necrophorum* n = 1.

***Haemophilus influenzae n = 6, *Pasteurella multocida* n = 5, *Capnocytophaga canimorsus* n = 1, *Francisella tularensis* n = 1, Lactobacillus species n = 1, *Moraxella catarrhalis* n = 1.

EUCAST guidelines and limit were used to define sensitive or resistant.

A statistically significant difference in overall susceptibility to the antibiotics used was found (8.4% resistance in the monotherapy group and 3.0% in the group receiving SGA, *p* = 0.021).

### Differences in treatment and mortality

The eight different β-Lactam antibiotics used for the initial treatment are presented in Table 3. The antibiotics were grouped into broad spectrum (β-lactams with antipseudomonal effect) (ceftazidime, imipenem/cilastatin, meropenem and piperacillin/tazobactam) and other β-lactams (benzyl-penicillin, cefotaxime, cefuroxime and cloxacillin).

**Table 3:** Initial β-lactam treatment and 28-days mortality comparison between study groups.

	β-lactams	β-lactam monotherapy n = 202		SGA-group^1^ n = 197		β -Lactam monotherapy vs SGA groups			
						Unadjusted		Adjusted^2^	
		n	Mortality^3^ (%)	n	Mortality^3^ (%)	HR (95% CI)	*p*	HR (95% CI)	*p*
Broad-spectrum β-lactams	Total	77	20 (26)	30	1 (3)	8.6 (1.1–64.2)	0.036	10.2 (1.3–76.9	0.024
	Ceftazidime	6		2					
	Imipenem/cilastatin	16		7					
	Meropenem	6		1					
	Piperacillin/tazobactam	49	12 (24)	20	0 (0)	n/a^4^	0.014	n/a^4^	
Other β-lactams	Total	125	25 (20)	167	167 (11)	1.8 (1.0–3.3)	0.045	3.5 (1.8–6.8)	<0.001
	Benzylpenicillin	20	3 (15)	60	3 (5)	3.3 (0.7–16.3)	0.15	29.4 (2.6–335)	0.006
	Cefotaxime	94	19 (20)	105	16 (15)	1.3 (0.7–2.6)	0.38	2.2 (1.0–4.6)	0.038
	Cefuroxime	5		1					
	Cloxacillin	6		1					
									

^1^ SGA in combination with a β-lactam antibiotic.

^2^ Adjusted for age, Charlson comorbidity score, SOFA-score and NEWS2-score as continuous variables and sex and immunosuppression as categorical variables.

^3^ 28-days mortality in number and percentage.

^4^ Not applicable due to no mortality cases in the SGA-group.

In the SGA-group, broad spectrum was used in 15% (30/197) and in the β-Lactam monotherapy group 38% (77/202), *p*<0.001.

In the subgroup treated with broadspectrum β -Lactam the adjusted HR for mortality was 10.2 (95% CI 1.3–76.9), *p* = 0.024 in favour for the SGA and in the subgroup of patients treated with other β-lactams adjusted HR was 3.5 (95% CI 1.8–6.8), *p*<0.001.

Among patients treated with the most common β –Lactams (cefotaxim, benzylpenicillin and piperacillin/tazobactam) the same pattern were seen.

### Acute Kidney Injury (AKI)

After excluding 107 patients with CKD, 292 patients were evaluated for AKI based on the change in s-creatinine. S-creatinine was registered at four time points. Nine patients at T2 and 22 patients at T3 had died and four patients at T2 plus 10 patients at T3 had been sent home. At admission, 92 patients had higher s-creatinine than at baseline (T0). Thirty-six patients had s-creatinine at Risk level according to RIFLE. Injury occurred in 38 patients and Failure in 18 patients. Of the 292 patients, 146 were in the cohort who received an addition of SGA.

**Fig 3:**
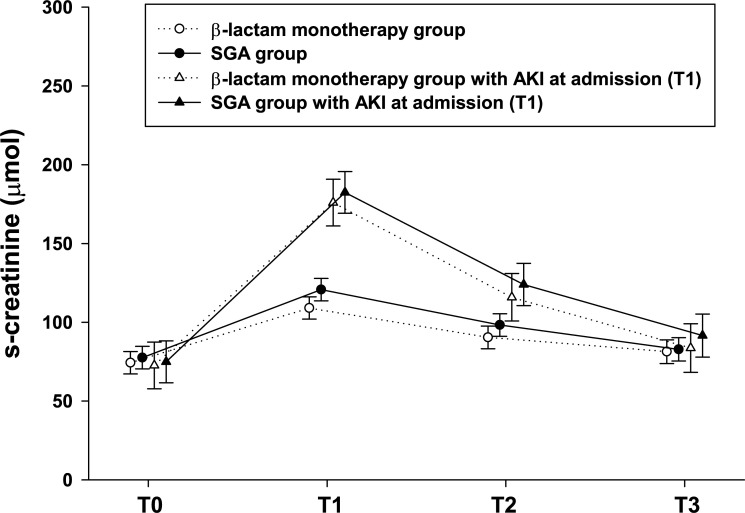
AKI. Mean creatinine from T0 to T3 in β-lactam monotherapy group and SGA group as well for each study group with only patients with AKI at admission (T1). Estimated mean creatinine with 95% confidence intervals by linear mixed model, see statistical section for details.

The unadjusted mixed model showed that the SGA-group (n = 146) had a mean s-creatinine of 121 μmol (95%CI 114–128) at T1, which decreased statistically significantly to means of 98 μmol (95% CI 91–106) at T2 and 83 μmol (95% CI 75–90) at T3 (Fig 3). The β-lactam monotherapy group (n = 146) had a mean creatinine of 109 μmol at T1, which decreased significantly to means of 90 μmol (95% CI 83–98) at T2 and 81 μmol (95%CI 74–89) at T3. From T1 to T2 as well as from T1 to T3, the mean change between the study groups was not significant, p = 0.51 resp. p = 0.08.

Among patients with AKI at T1 (n = 51 SGA group and n = 41 β-lactam monotherapy group), the mean change of s-creatinine between study groups from T1 to T2 was unadjusted 2 μmol (95% CI −21 to 24), *p* = 0.88, and adjusted 2 μmol (95% CI −20 to 24), *p* = 0.85 (Fig 3). Of the 92 patients with AKI at T1, 9 still had increased s-creatinine at T3 (6 in the SGA group and 3 in the β-lactam monotherapy group), *p* = 0.49. No altered statistical findings were found in the adjusted models.

Fourteen patients with normal s-creatinine at admission (T1) had developed AKI at T3. Of these 14 patients, 8 were in the SGA group and 6 in the β-lactam monotherapy group (*p* = 0.62).

In patients with sepsis but no shock, 67 had AKI at T1 (36 in SGA group and 31 in β-lactam monotherapy group, *p* = 0.42) compared with 25 patients with septic shock (15 in the SGA group and 10 in the β-lactam monotherapy group, *p* = 0.27). AKI was more frequent in patients with septic shock (46%, 25/54) than in patients with sepsis but no shock (28%, 67/238), *p* = 0.010.

### Missing data

Bilirubin and platelet blood count were not routinely used at all the participating departments at the time of the study. To ensure that this would not be a potential bias, OR for mortality in the group of patients with complete SOFA scores (n = 327) was calculated: unadjusted HR 2.6 (95% CI 1.4 to 4.6), *p* = 0.002 and adjusted HR 4.2 (95% CI 2.1 to 8.2), *p* < 0.001 in favour of the addition of SGA. Concerning AKI, some patients died before creatinine follow-up (T2 and T3), which also resulted in missing data.

## Discussion

This study was a retrospective observational study conducted over two years including 399 patients with sepsis and positive blood cultures. The aim of the study was to evaluate the effect on mortality and safety regarding kidney function after adding one SGA to a β-Lactam antibiotic in the treatment of patients with community-acquired bloodstream infection.

The 28-day mortality was significantly lower in the group receiving the addition of SGA in all groups of patients independent of potential confounding variables. Several studies have previously analysed the addition of aminoglycosides to β-lactam antibiotics in the treatment of sepsis. Many of these were included in a recent Cochrane review concluding that the addition of an aminoglycoside did not affect 28-day mortality. Importantly, the Cochrane report did not include any study in which only one single dose of an aminoglycoside was administered at admission, but only studies that used single daily doses or multiple daily doses over several days and studies including patients with both community-acquired or nosocomial infections. The authors concluded that the use of β-Lactam and gentamicin combination therapy for sepsis should be discouraged [11]. Conversely, Kumar et al. showed significant benefit in mortality outcome with combination therapy for patients with septic shock [12], but in patients with sepsis without shock, it rather showed adverse effect with increased mortality [13]

The present study showed no higher risk of developing AKI in the SGA group than in the β-lactam monotherapy group. Previous sepsis studies have investigated patients receiving a course of gentamicin once daily or multiple doses daily [11]. Saturated tubular cells in the renal cortex are suggested to be targets for toxic effects of gentamicin and therefore a possible cause of AKI [17, 35]. By adding only one single dose, the patients still gain the advantage of a wider antibiotic spectrum [14, 15, 36, 37] but the tubular cells may be spared from saturation, and might not develop AKI. The results of this study are in line with Cobussen et al. [23], who also reported that a single dose of gentamicin does not result in increased risk of AKI. This suggests that AKI might be caused not only by the administration of gentamicin to patients with sepsis or septic shock but perhaps also by other confounding factors such as hypovolemia, comorbidities, or the organ dysfunction caused by sepsis itself [38].

The present study was a retrospective observational study, including only adult patients with community-acquired bloodstream infections fulfilling the Sepsis-3 criteria for sepsis or septic shock. Nosocomial infections with bloodstream infections were excluded to refine the study population, since these patients may had been through surgery (with or without complications) or may had received prior antibiotic treatment. Subsequently this could induce different Anti-Microbial Resistance (AMR) profiles from nosocomial pathogens in that higher levels of resistance are likely to be encountered [39–41].

It evaluated the effect of adding one SGA to β-Lactam directly after cultures were secured at the ED with an emphasis on limiting the administration to one single dose of gentamicin. These data could motivate clinicians to use combination therapy with gentamicin and β-Lactam antibiotics as an alternative treatment in countries with low rates of AMR despite the earlier described risk of nephrotoxicity.

The results from this study are in contrast to the meta-analysis by Paul et al. [11]. We have tried to adjust for confounding variables but still we notice a benefit of SGA. The metanalysis [11] included a range of studies with different designs, patient populations, antibiotic agent combination therapies, bacterial spectra, studies includingboth community acquired infections and nosocomial infections and maybe most importantly no studies using the SGA approach as performed here.

The study presented here has some limitations. First, and most important, it is a retrospective, observational study, which inhibits conclusive assessment of the causality between improved mortality outcome and combination antibiotic therapy. Second, eight different β-Lactam antibiotics with varying antibiotic spectrum were used, four of which were broad spectrum. This makes it difficult to statistically compare them further since each sample size is limited. Previousarticles [42–44] have described increased risk of ototoxicity after administration of an aminoglycoside. This could not be evaluated in this study since the patients’ hearing was not measured. Moreover, since most information on potential risk factors such as smoking, excessive use of alcohol, and patient delay were lacking in a majority of the medical charts, these factors were not evaluated.

However, this one-centre study included 399 patients with few missing data, which gives strength to the results. Despite the use of sophisticated methods to account for individual patient differences, observational studies may be confounded by indication. Gentamicin might be less frequently administered to patients with CKD, but no significant difference in the prevalence of CKD was found between the two groups. We also only investigated patients with sepsis and positive blood culture and we can thus not draw any conclusion of the effectiveness of the addition of SGA in patients with sepsis despite negative blood culture.

There is a possible synergistic effect between β-Lactam antibiotic and gentamicin. The synergistic effect can be attained by β-Lactam inhibiting bacterial cell-wall synthesis and lysis and simultaneously aiding the transport of gentamicin through the cell membrane where it binds to the ribosomes causing mistranslation and defective protein synthesis [45, 46] and has been demonstrated in vitro [47, 48]. Other studies have proposed other theories such as a reduced release of endotoxins [49] or an influence on oxidative burst and oxidative stress as a consequence of increased reactive oxygen radicals in the cytoplasm of bacteria [46]. The mechanism behind these results were beyond the scope of this study and parts of the positive effects seen with the addition of SGA were in many cases attributed to the widened antibacterial spectrum obtained with the combination.

## Conclusion

This retrospective observational study including patients with community-acquired sepsis or septic shock and positive blood cultures, who meet Sepsis-3 criteria, shows that the addition of one single dose of gentamicin to β-lactam treatment at admission was associated with a decreased risk of mortality and was not associated with AKI. Further prospective studies are warranted to confirm if this antibiotic regime may be an alternative to the use of broad-spectrum β-lactam antibiotic treatment of community-acquired sepsis.

## Supporting information

S1 File (XLSX)

S2 File (DOCX)
