# Supplementary material for: Antibiotic treatment with one single dose of gentamicin at admittance in addition to a β-lactam antibiotic in the treatment of community-acquired bloodstream infection with sepsis
Source: PLoS One. 2020 Jul 30;15(7):e0236864. doi: 10.1371/journal.pone.0236864 (PMC7392313; doi:10.1371/journal.pone.0236864)
Supplement: S2 File — (DOCX) [file pone.0236864.s002.docx]

**Supplement 1.**

**Intensive care unit**

Sixty-seven patients, 37 men (55%), mean age 72.0 years were admitted to the ICU for treatment. Most common bacterial agents were e-coli (22%), Staph. Aureus (21%) and pneumococci (16%). About half (53%) received an addition of aminoglycoside at admittance to the hospital and 28 (42%) had AKI at T1 (admittance). All patients had at least one organ failure. Most common was respiration failure (91%), kidney failure (67%) or coagulation difficulties (40%).

The patient group had Charlson index median of 2, IQR 1-3, immunosuppression in 15 patients (22%) and mortality 14 (21%).

***Staphylococcus Aureus* infections**

Seventy-four patients, 53 men (72%) with mean age 74.1 years. 14 (19%) patients were admitted to the ICU and total mortality was 22%. AKI was detected in 15 (20%) patients at T1. Most common diagnosis was uncertain focus of Staphylococcus Aureus sepsis 55 patients (74%), endocarditis 10 patients (14%), cutaneous abscesses in 6 patients (8%) and spondylodiscitis 3 (4%).

**Immunosuppressed patients**

A total of 51 patients had some sort of immunosuppression (see table 1). Twenty three men (45%), mean age was 71.3 and Charlson index median was 2, IQR 1-4. AKI appeared at T1 in 12 patients (24%). Most common agent were E.-coli 16 patients (31%), Streptococci 13 (26%), Staph. Aureus 7 (14%) and Pseudomonas 2 patients (4%).

**Supplement 2.**

**Table 4.** Risk of mortality in patients receiving β-lactam monotherapy versus patients receiving β-lactam together with one single dose of gentamicin at admission (SGA).

|  |  | Mortality  (28-days) | Unadjusted | | Adjusted^1^ | |
| --- | --- | --- | --- | --- | --- | --- |
|  | **n** | **no. (%)** | **HR (95% CI)** | ***p*** | **HR (95% CI)** | ***p*** |
| **Study groups** |  |  |  |  |  |  |
| β-lactam monotherapy | 202 | 45 (22.3%) | 2.3 (1.4-3.9) | 0.002 | 3.5 (1.9-6.2) | <0.001 |
| SGA-group^2^ | 197 | 20 (10.2%) | reference |  | reference |  |
| **Sex** |  |  |  |  |  |  |
| Female | 183 | 25 (13.7%) | reference |  | reference |  |
| Male | 216 | 40 (18.5%) | 1.4 (0.8-2.3) | 0.18 | 1.5 (0.0-2.5) | 0.11 |
| Age, per year |  |  | 1.04 (1.02-1.06) | 0.001 | 1.06 (1.03-1.08) | <0.001 |
| Charlson Score, per unit |  |  | 1.21 (1.07-1.37) | 0.003 | 1.07 (0.94-1.22) | 0.31 |
| SOFA-score, per unit |  |  | 1.28 (1.15-1.42) | <0.001 | 1.26 (1.12-1.43) | <0.001 |
| NEWS2-score, per unit |  |  | 1.14 (1.05-1.23) | 0.001 | 1.10 (1.01-1.20) | 0.026 |
| Immunosuppression |  |  |  |  |  |  |
| No | 348 | 57 (16.4%) | reference |  | reference |  |
| Yes | 51 | 8 (15.7%) | 0.9 (0.4-2.0) | 0.88 | 1.0 (0.4-2.1) | 0.96 |
| β-Lactam |  |  |  |  |  |  |
| Broad-spectrum | 107 | 21 (19.6%) | 1.3 (0.8-2.2) | 0.34 | 0.9 (0.5-1.7) | 0.83 |
| Other | 292 | 44 (15.1%) | reference |  | reference |  |

^1^Adjusted for age, Charlson comorbidity score, SOFA-score and NEWS2-score as continuous variables and sex, broad- or narrow antibiotic spectrum and immunosuppression as categorical variables.

^2^ SGA in combination with a β-Lactam antibiotic

HR = Hazard Ratio

CI= Confidence Interval
